# Review of the Impact of Apple Fruit Ripening, Texture and Chemical Contents on Genetically Determined Susceptibility to Storage Rots

**DOI:** 10.3390/plants9070831

**Published:** 2020-07-02

**Authors:** Hilde Nybom, Masoud Ahmadi-Afzadi, Kimmo Rumpunen, Ibrahim Tahir

**Affiliations:** 1 Department of Plant Breeding–Balsgård, Swedish University of Agricultural Sciences, Fjälkestadsvägen 459, 29194 Kristianstad, Sweden; kimmo.rumpunen@slu.se; 2 Department of Biotechnology, Institute of Science, High Technology and Environmental Sciences, Graduate University of Advanced Technology, Kerman 7631818356, Iran; m.ahmadiafzadi@kgut.ac.ir; 3 Department of Plant Breeding, Swedish University of Agricultural Sciences, Box 101, 23053 Alnarp, Sweden; ibrahim.tahir@slu.se

**Keywords:** *Botrytis cinerea*, *Colletotrichum*, disease resistance, *Malus* × *domestica*, *Monilinia*, *Neofabraea*, *Penicillium expansum*, plant breeding

## Abstract

Fungal storage rots like blue mould, grey mould, bull’s eye rot, bitter rot and brown rot destroy large amounts of the harvested apple crop around the world. Application of fungicides is nowadays severely restricted in many countries and production systems, and these problems are therefore likely to increase. Considerable variation among apple cultivars in resistance/susceptibility has been reported, suggesting that efficient defence mechanisms can be selected for and used in plant breeding. These are, however, likely to vary between pathogens, since some fungi are mainly wound-mediated while others attack through lenticels or by infecting blossoms. Since mature fruits are considerably more susceptible than immature fruits, mechanisms involving fruit-ripening processes are likely to play an important role. Significant associations have been detected between the susceptibility to rots in harvested fruit and various fruit maturation-related traits like ripening time, fruit firmness at harvest and rate of fruit softening during storage, as well as fruit biochemical contents like acidity, sugars and polyphenols. Some sources of resistance to blue mould have been described, but more research is needed on the development of spore inoculation methods that produce reproducible data and can be used for large screenings, especially for lenticel-infecting fungi.

## 1. Introduction

Although apple *Malus* × *domestica* is one of the most important and well-studied fruit crops in the world, growers still face a number of unresolved problems with production, storage and marketing. A major proportion of the commercially grown fruit is intended for marketing after a period of at least four months in cold storage. However, fungal decay during storage leads to considerable losses of fruit. The situation is especially serious in low-input production systems (like organic orchards and agroforestry systems), where the fruit cannot be adequately protected by fungicides and losses can amount to 20 times as high as those in conventional orchards [1,2,3]. Grower revenues are often reduced since the fruit must be marketed after a shortened storage period.

The number of approved fungicides has decreased in conventional production systems, and postharvest applications are completely banned in an increasing number of countries [4], while treatment with the protective gas 1-MCP (1-methylcyclopropene) is avoided, e.g., in Scandinavia, for marketing reasons, thus emphasizing the need for apple cultivars with a higher resistance. As a consequence, we now see an increasing number of reports on the relative levels of resistance/susceptibility to various storage rots. Variation among cultivars in this respect may be associated with phenotypic and physiological traits like fruit-ripening behaviour, including changes in internal ethylene content (IEC), fruit texture, fruit epidermis structure and chemical contents like sugar and various antifungal components such as chlorogenic acid and quercetin [4,5,6,7,8,9,10,11,12,13,14,15]. 

In this review, methods for quantifying damage caused by various storage rots are described, and information is provided about the impact of some fruit-ripening-related traits on susceptibility to storage rots. Hopefully, this will be valuable for plant breeders aiming to produce cultivars with an improved ability to withstand attacks of storage rots. 

## 2. Fungi That Cause Storage Rot

A number of ascomycete fungi, often known as storage rots, regularly attack apples both in the orchard and during cold storage (Table 1). Symptoms are first visible as lesions on the fruit epidermis and can proceed to rotting of the entire fruit. Some of the most important storage rots in apple belong to two biotrophic or hemibiotrophic genera and are also known as latent or quiescent infection pathogens, namely *Neofabraea* (e.g., *N. alba* (syn. *N. vagabunda)*, *N. kienholzii*, *N. malicorticis* and *N. perennans*) and *Colletotrichum* (the most well-known being *C. acutatum* and *C. gloeosporioides,* both of which have been split into several taxa), while other important species include the mainly necrotrophic (‘wound pathogens’) *Penicillium expansum*, *Botrytis cinerea*, *Monilinia fructigena* and *M. laxa* [16,17,18,19] (Table 2). In addition, numerous fungi that can infect apple blossoms and cause large necrotic areas on the fruit are known as blossom-end rots or calyx-end rots [20]. Relative importance of these and other fungi, e.g., *Alternaria* spp., *Botryosphaeria obtusa*, *Cadophora luteo-olivacea*, *Fusarium* spp., *Mucor pyriformis*, *Neonectria ditissima* and *Phacidiopycnis washingtonensis*, varies widely between geographic areas as well as from year to year.

The very common disease bull’s eye rot, sometimes also known as lenticel rot, is caused by *Neofabraea* spp. These fungi infest the fruit during the growth phase in the orchards, from petal fall to harvest, with susceptibility increasing gradually during the ripening process [25]. The spores remain dormant in the lenticels and start to grow only when the fruit has reached a certain stage of maturity, probably due to physiological changes associated with a decline in fruit antifungal compounds or defence responses [42]. The germinating conidia form hyphae that penetrate the epidermis of the fruit, preferentially through open lenticels but also through the stem bowl and calyx-end regions, or through microcracks on the surface [22]. Bitter rot, caused by *Colletotrichum* spp. has a similar mode of infection [67]. Symptoms of the lenticel-infecting fungi usually do not appear until the fruit has been kept in cold storage for some weeks and thereafter at room temperature for some days. Moreover, storage-rot-producing species of *Neofabraea* can also cause perennial cankers (anthracnose) on apple trees, thus ensuring year-round survival of the pathogen in the orchard [46], while species of *Colletotrichum* are responsible for the widely dispersed Glomerella leaf spot disease (named after the sexual stage of *C. gloeosporioides*, namely *Glomerella cingulata*) [35,36]. 

The most well-studied of all storage rots to date is *P. expansum* (blue mould), which causes serious damage to apple crops all around the world. This fungus also produces the mycotoxin patulin, which is very harmful to human health and occurs as a contaminant especially of apple juice and unfermented apple cider [68,69]. Infections by *P. expansum,* as well as by some other storage rots like *B. cinerea* (grey mould) and *M. fructigena* (brown rot), are mainly wound-mediated. The hyphae enter through wounds, e.g., caused by birds and insects or the inattentive handling of the fruit during harvest, storage and transportation, but infection can also take place through an open calyx of the flower [25]. Symptoms are sometimes visible already in the orchards, but develop mainly during storage.

## 3. Quantification of Storage Rot Damage

Genetic variation among cultivars (each cultivar usually corresponds to a single genotype in apple and other clonally propagated crops) is the basis for selection-mediated improvement, and the subsequent development of superior cultivars. Considerable variation among cultivars in their susceptibility to some of the most common storage rots has been reported from observations in orchards and storage rooms [3,19,21,40,43,70]. Still, levels of resistance cannot be properly quantified based only on natural infections, since the inocula may vary in both quantity and virulence. There are also problems with attributing visible damage to a particular fungal species since symptoms on the fruit may stem from several fungi. Taxonomically correct identification of the involved fungi can be difficult, and morphological evidence may have to be complemented with time-consuming molecular analyses [16,21,44].

In order to obtain properly quantifiable data for a large set of genotypes, experimental inoculations with well-defined inocula have been carried out for several fungi (Table 3). For the wound-mediated storage rots like *P. expansum*, *B. cinerea* and *M. fructigena*, inoculation is usually achieved by wounding and inoculating a number of fruits with conidiospores of the fungus [5,8,15,21,24,25,31,49,56,57]. Wounding and inoculation can be carried out simultaneously, using a pipette with disposable plastic pipette tips to create one to three inoculation sites on each fruit, allowing large numbers of fruit to be processed in a limited amount of time. Disease severity is usually assessed as the diameter of each lesion, measured at a predefined point in time after a period of storage. A lesion expansion growth rate (LEGR) can be calculated by linear regression if repeated measurements are made over several days or weeks [28]. In addition to the most commonly used parameter, i.e., lesion diameter = disease severity (S), the percentage of inoculations producing a lesion can be quantified as disease incidence (I). Infection severity is sometimes calculated as (I × S)/100. However, I and S were closely correlated in a study on wild accessions of *M. sieversii* that had been wound-inoculated with *P. expansum* [56].

The wound-inoculation method has been applied also for fungi that usually enter through the lenticels, like *Colletotrichum* [5,27,30,31] and *Neofabraea* [25,48,49]. Conidiospores for the inoculum can be more difficult to produce for some of these species compared to *P. expansum*, and inoculation success may be somewhat lower [5]. Additionally, contamination by faster growing fungi, like *P. expansum*, can become quite problematic.

Since wound-inoculation data cannot provide an estimate of the ease with which lenticel- and micro-crack-infecting fungi enter the fruit, inoculum is sometimes instead sprayed onto the fruits, either while still growing on the tree [46,50] or after harvest [30,43]. Dipping the entire fruit into a spore solution has also been practiced [8]. Dipping and spraying often produce several infections on the same fruit, but symptom development is slower and less consistent compared to wound inoculations [30]. The resulting symptoms are usually quantified as number of lesions or as relative affected area, but the obtained high standard deviations may render this method unfit for comparisons of cultivar susceptibility [30]. In another study, spores of *N. alba* were placed on water agar, and fruits were placed on these spores in a Petri dish for at least 7 days to establish an infection [28]. Reliable infection of 66–100% of the fruits required 14 days of contact. A similar approach was used also for *C. acutatum*, with a wetness period of 72 h required for lesion development [67].

In a comparative study, Biggs and Miller [27] inoculated a set of 18 apple cultivars with *C. acutatum* using three methods: (1) dressing fruits in the field 3–4 weeks pre-harvest with cheesecloth strips soaked in a spore solution, (2) wound-inoculation of fruit harvested 2–3 weeks before their normal harvest date, and (3) attachment of a microcentrifuge tube with a spore suspension onto an unwounded surface of the very same fruits as used in the wound inoculations. The third method, which was quite labour-intensive, showed the highest reproducibility between the two years of experiments; both disease incidence and disease severity (lesion size) produced meaningful data.

All the methods described above are based on inoculation with conidiospores, but some studies have instead utilized mycelia. Mycelial discs (7 mm diameter) were cut with a cork borer from the edges of actively growing cultures of *Botryosphaeria dothidea*, and five discs were then taped onto a detached fruit [22]. Similarly, small pieces of mycelia from *N. alba* have been inserted as plugs into fruit flesh, or placed in pockets under the skin of the fruit [40,45]. In a slightly modified version, fruit pulp plugs (5 × 3 mm) were extracted from detached fruit and replaced with equal-sized mycelial plugs of 21-day old *Neofabraea* cultures, covered by moist cotton and sealed with Parafilm [39]. Significant differences in lesion size were found among the inserted fungal isolates, but not between the two apple cultivars. Mycelial plugs have also been used for inoculation with *Botrytis cinerea* [26]. These methods are, however, considerably more time-demanding than the spore-based methods, and have not yet been used for screening large sets of cultivars/genotypes.

The inoculated fruit is often kept at room temperature while infections develop. Lesions usually appear within a few days, and the damage can be scored within a week after inoculation, although slow-growing fungi like *Neofabraea* may require incubation for 15 days [39] or a whole month before symptoms have developed sufficiently [28]. In one approach, some fruits were wound-inoculated with *Colletotrichum* either at harvest or after three months of cold storage [30]. The inoculated fruits were subsequently kept at room temperature until evaluation of symptoms for a period of 4 to approximate 20 days. The lesions derived from inoculation of previously cold-stored fruit developed faster and showed a higher cultivar effect, presumably because the fruits were more mature when infected.

In studies aiming to compare large sets of cultivars for their innate susceptibility to storage rots, commercial cold-storage conditions are often applied during disease development [5,15,25]. In studies of lenticel-infecting fungi like *Neofabraea*, cold storage is usually followed by 5–7 days at room temperature in order for symptoms to develop properly. In a broad screening, the number of weeks in cold storage may need to be adjusted for ripening period of the cultivars; early-ripening cultivars (‘summer varieties’) can usually be stored only for a few weeks before major decay due both to fungi and other factors sets in. By contrast, late-ripening cultivars (‘fall and winter varieties’) may have to be stored for 10 weeks or longer in order for lesions to develop properly. For comparison of cultivars stored for different lengths of time, a lesion decay index can be obtained by dividing the average lesion diameter by number of weeks in storage [15].

Great care must be taken not only in the inoculation and storage procedures, but also in the origination of the fruit to be tested. Ideally, the harvested trees should have been grown in the same orchard and have a similar age, since fruit on younger trees are more susceptible to infections such as *N. malicorticis* [71]. The fruits should also be harvested at the same maturity stage; fully mature fruit are more susceptible to various storage rots [4,41,53,56,59,67,72,73,74]. Since apple cultivars differ widely in ripening period, the inoculations have to be staggered in time so that all genotypes are inoculated at an optimal stage. Ideally, the inoculations should be repeated over several years, as fruit-ripening parameters are very sensitive to weather fluctuations. Fortunately, wound-based inoculations with *P. expansum* [5,15] and with *C. acutatum* [27] show reasonably high reproducibility between years. Even higher reproducibility was, however, obtained with attached microcentrifuge tubes, whereas field inoculation with cheesecloth strips produced data that were not correlated with the laboratory methods [27].

In addition to producing reproducible data, an efficient inoculation method must also be able to differentiate properly among a large number of cultivars. Significant variability among cultivars has been reported after wound-inoculation of harvested fruit with *P. expansum* [5,15,25,58,60]. Some well-known commercial cultivars, e.g., ‘Fuji’, ‘Gala’, ‘Gloster’, ‘Katja’/‘Katy’ and ‘Mutsu’, appear to be relatively resistant, as do some locally grown cultivars, e.g., the Chinese ‘Fu Shuai’, Latvian ‘Olga’ and Russian ‘Pepin Schafranovij’. Interestingly, some genotypes of the apple progenitor species *M. sieversii* appear to be highly resistant [31,52,56,57]. Wound inoculation with the wound-mediated fungus *B. cinerea* has similarly produced significant differences among cultivars [8,25]. This method has also been successfully applied for lenticel-infecting fungi like *N. malicorticis* [25], *N*. sp. (‘*Gloeosporium fructigenum*’ [8]) and *C. acutatum* [5,27,31]. In a comparative study, wound inoculation with *C. fioriniae* was deemed to be more suitable for research purposes than spraying, since symptoms on sprayed fruit were difficult to quantify [30]. Unfortunately, it is presently not possible to identify cultivars with above-average resistance to fungi other than *P. expansum*, as screenings for those fungi have generally included a very restricted number of genotypes.

## 4. Resistance Mechanisms and Quest for Resistance Genes

For the biotrophic and lenticel-infecting fungi, the number of lenticels and thickness of the cuticular layer of the fruit are crucial factors, as shown in a study of 11 apple cultivars colonized by *Botryosphaeria dothidea*, which causes apple ring rot in Asia [22]. Similarly, an open calyx in the flower can provide an entry point for several storage rots (Table 2). For both biotrophic and necrotrophic fungi, infection ability is also dependent on the complex interaction between the fungi and their hosts in sophisticated recognition and signalling networks. A common response of a plant after fungal attack is the accumulation of reactive oxygen species (ROS) known as an oxidative burst. This can have a detrimental effect on the pathogen directly, damaging the plasma membrane and mitochondrial proteins, but can also activate various defence pathways in the attacked plant [57,66]. The plant then needs to remove excess ROS in order to protect itself, which is usually accomplished via an increase of ascorbate and glutathione. The fungal attack proceeds by a production of various compounds that can overcome the innate immune system of the host, including phytotoxic compounds, cell-wall-modifying enzymes and proteinaceous effectors. These changes have complex effects on several different substances in the fruit. Content of total phenols thus increased shortly after inoculation with *P. expansum* in one study, possibly due to activation of phenylalanine-ammonia lyase, and then decreased again [38]. Žebeljan et al. [66] observed a decline in fructose, malic acid, shikimic acid and total ascorbate content six hours post infection (hpi) with *P. expansum*. This was followed by a significant increase in malic acid at 24 hpi, and in total glutathione three days post infection (dpi). Finally, at 5 dpi, there was a significant increase in sucrose together with a decline in glucose and ascorbate.

Similar studies have been carried out after inoculations with *B. cinerea*. The susceptible cultivar ‘Braeburn’ initially had a higher content of ascorbate in the peel and of other antioxidants in the fruit flesh after infection with this fungus, together with a more pronounced oxidative burst after two weeks, in comparison with the almost completely resistant ‘Golden Delicious’ [23]. By contrast, infection with *B. cinerea* produced an increase in chlorogenic acid in the relatively resistant cultivar ‘Qinguan’, but a decrease in the more susceptible ‘Fuji’ [24]. Several enzymes, including phenylalanine-ammonia lyase, also showed higher activity in ‘Qinguan’ compared to ‘Fuji’. Obviously, associations between contents of various antioxidants and levels of enzyme activity on the one hand, and degree of resistance/susceptibility on the other hand, are not clear-cut.

Pectin degradation of cell walls is part of the fruit-ripening process in apples, and cell-wall degradation can be hastened through the secretion of lytic enzymes by the fungus, like cellulase and especially xylanase [43]. Unripe apples have relatively low pH (reaching below pH = 4 in some cultivars), but pathogens can decrease or increase this value. *Penicillium expansum* lowers the pH of its host via production of various organic acids (especially gluconic acid), thus providing an optimal environment for the cell-wall-degrading enzymes [12,54]. Interestingly, mutant strains of *P. expansum* that lack the global carbon catabolite regulator (CreA) cannot produce patulin and are also avirulent, due to a decreased ability to produce proteolytic enzymes and to acidify the *in planta* environment [62].

Among the common defence pathways of the plant, a major role has been indicated for jasmonic acid- and ethylene-mediated defence responses, as well as the phenylpropanoid metabolism [24]. Application of the ethylene-mediating plant hormone methyl jasmonate (MeJA) onto apples enhances expression of several ethylene-related genes like *MdACS1* (1-aminocyclopropane-1-carboxylate synthase) and *MdACO1* (1-aminocyclopropane-1-carboxylate oxidase) [75,76]. Both of these genes are associated with inter-cultivar variation in fruit firmness at harvest and after storage [77,78], which appears to be an important component in the resistance against storage rots. Application of n-propyl dihydrojasmonate similarly induced ethylene production and an increased activity of several ethylene-related genes, and also resulted in smaller lesions on apples inoculated with *B. cinerea* [26].

The importance of these pathways has been corroborated by gene expression studies. A whole-genome apple microarray with 60 thousand transcripts was used to identify genes in *P. expansum*-infected fruits of four apple cultivars, two less susceptible and two very susceptible [51]. Transcriptomic analyses of fruit sampled 1 week and 6 weeks after inoculation produced large differences between the very susceptible and the more tolerant cultivars. Potential candidates were identified among defence- and oxidative-stress-related genes, cell-wall modification and lignification genes, and genes related to localization and transport. The fundamental role of the cell wall as an important barrier was demonstrated by induction of a cell-wall-related gene. Similarly, three genes involved in the ‘downstream’ flavonoid biosynthesis pathway were implicated as being important for resistance. In addition, exogenous application of MeJA reduced the symptoms resulting from inoculating fruit with *P. expansum*. A comparison between a more resistant genotype of *M. sieversii* and the susceptible *M.* × *domestica* cultivar ‘Royal Gala’ was carried out on fruit sampled 0–48 h after inoculation with *P. expansum* [52]. As expected, gene expression analysis suggested a higher basal level of resistance in *M. sieversii* compared to that in ‘Royal Gala’, as well as a more rapid and intense defence response to wounding and to wounding plus inoculation. Again, ethylene-related genes and genes involved in the ‘jasmonate’ and ‘MYB-domain transcription factor family’ groups were overexpressed in the resistant genotype.

In a study based on inoculating apples with both *P. expansum* and the non-host pathogen *P. digitatum*, infection by *P. expansum* reduced the ethylene climacteric burst in the fruits together with induction of the *MdACO3* gene, together with downregulation of ACO (1-aminocyclopropane-1-carboxylic acid oxidase) enzyme activity and overexpression of ACS (1-aminocyclopropane-1-carboxylic acid synthase) activity [64]. The authors hypothesized that *P. expansum* ‘manipulates’ the endogenous ethylene biosynthesis in apples, leading to the circumvention or suppression of effective defences against the fungus. In another study, fruits infected by *P. expansum* showed higher peaks in both ethylene content and respiration compared to uninfected fruits, together with reduced membrane integrity and fruit firmness [54].

Wang et al. [65] focused on just the first six hours after infecting apples with *P. expansum*. The main differentially expressed genes identified in *P. expansum* were related to cell-wall-degradation enzymes, anti-oxidative stress, pH regulation and effectors. The host responded by activating pathogen-associated molecular pattern (PAMP)-triggered immunity (PTI) one hour after infection, and effector-triggered immunity three hours after infection. In another gene expression study of *P. expansum,* genes coding for pectin degradation were again shown to be upregulated during infection of apple [79]. Barad et al. [32] showed that gene expression patterns of *P. expansum* are, however, extremely versatile; some genes expressed during infection of apple were pH neutral, while others were similar to those obtained when growing the fungus in vitro at pH 4 or at pH 7, respectively. In the same study, infected apple tissue responded by upregulating genes involving jasmonic acid, mevalonate and flavonoid biosynthesis pathways, but the response was stronger towards *P. expansum* than towards *C. gloeosporioides*.

Virulence mechanisms differ widely between different fungi, and the involved compounds and pathways vary accordingly. Some fungi like *C. gloeosporioides* produce large amounts of ammonia during penetration and necrotrophic colonization of apples, thus raising the pH to over 8 [14]. The fungus-mediated pH changes are affected by genetically determined inter-cultivar variation in sugar content during ripening and at the final stage of maturity, and therefore also have an impact on the level of susceptibility [33]. For the lenticel-infecting fungi, the mechanisms by which the fungus can enter and then infect the fruits are of course critical. A gene expression study carried out in *C. fructicola* (belonging to the *C. gloeosporium* group) focused on the transcriptome in four types of fungal tissue, namely conidia, appressoria and infectious hyphae, sampled on inoculated apple leaves and in vitro using a cellophane membrane [34]. In the conidia, upregulated genes indicated a shunt towards triacylglycerol biosynthesis and thus an increased production of lipid droplets which constitute an important energy reserve. For penetration of the host tissue, melanization of the appressoria is a critical stage, as also reflected in the upregulation of genes involved in melanin biosynthesis. Growth inside fruits was not investigated, but growth of hyphae in apple leaves showed an upregulation of genes responding to phosphate starvation, thereby indicating a phosphate-depleted *in planta* environment.

Detection of genes with an impact on the resistance against storage rots has been attempted via different avenues. Traditionally, major genes and QTLs (quantitative trait loci) are identified based on a molecular-marker-mapped population with offspring that segregate for the trait of interest. A dense genetic map (3441 SNP markers) based on restriction-site-associated DNA sequencing (RADseq), enabled the location of QTLs controlling fruit firmness, sugar content and acidity in apples to be identified [80], i.e., traits that may have relevance for storage-rot resistance. Recently, inoculation of fruit harvested from a mapping population of ‘Royal Gala’ × *Malus sieversii* PI613981 resulted in the identification of two QTLs for resistance against *P. expansum* on LG3 and LG10, respectively [59]. The stronger of these, mapped from 67.3 to 74 cM on LG3 (qM-*Pe*3.1), explained 27.5% of the variation and appeared to derive from the *M. sieversii* parent. The second QTL, mapped to LG10, was presumably inherited from ‘Royal Gala’. Genes and QTLs for firmness and ripening have previously been found in the same region on LG10, and may be involved in this lower-level resistance.

Genome-wide association study (GWAS) is a more recent approach, which exploits the linkage disequilibrium present among individuals from natural populations or germplasm collections. These are usually more diverse than segregating progenies, and can be used to map QTLs with a higher resolution. In addition, the recent development of high-density SNP arrays with uniform coverage of the whole genome, makes it possible to obtain data for very large numbers of mostly anonymous markers. A 20K Infinium SNP Array (Illumina) was thus used in a GWAS to map QTLs for apple fruit volatiles, and to reveal their interplay with fruit texture by also scoring two functional markers, *MdPG1* (polygalacturonase 1), involved in the depolymerization of pectin, and *MdACO1*, catalysing the last step in the production of ethylene [81]. Interestingly, cultivars with a high aromatic volatile production usually had soft flesh, while cultivars with firm and crispy fruit had notably less aroma. Strong interplay between fruit-ripening-related traits of firmness and aroma are indicated; ethylene is known to affect the production of fruit volatiles, and a QTL for volatiles was also found in the same location as *MdPG1* on LG10.

The 20K Infinium SNP Array was also used in another fruit-texture analysis [82] with two approaches: GWAS on a collection of apple cultivars, and PBA (pedigree-based analysis) using six full-sib families. One QTL on LG10 appeared to determine fruit firmness while two QTLs on LG2 and LG14 were associated with crispness. GWAS have also been carried out using the Axiom^®^Apple 480K array developed within the Fruitbreedomics project [83]. Eight QTLs, two for flowering period and six for fruit-ripening period, could be mapped with high resolution using a total of 1168 apple genotypes [84]. Geographic origins and genetic relatedness among cultivars accounted for a large part of the phenotypic variation, suggesting that selection has been undertaken in response to the different growing environments.

**Figure 1:**
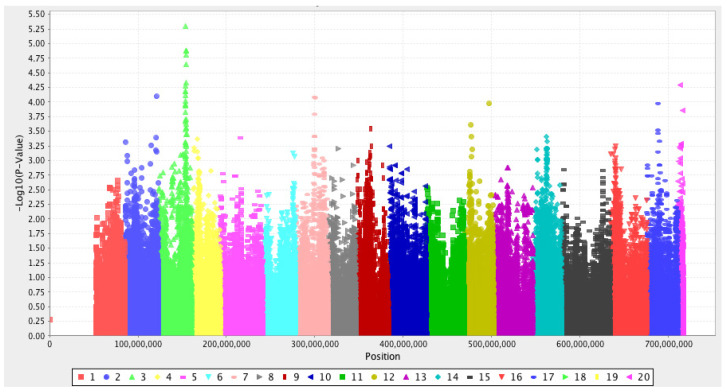
A Manhattan plot displays associations between data for lesion decay of 180 *Penicillium expansum*-inoculated apple cultivars and SNPs (single nucleotide polymorphism) in the Axiom^®^Apple 480K array for DNA samples of the same cultivars (Ahmadi-Afzadi, Muranty, Nybom, and Durel, unpublished). Genomic coordinates are shown along the *x*-axis with different colours according to which of the 17 apple-genome linkage groups each SNP is situated on, while the negative logarithm of the association *p*-value is displayed on the *y*-axis. A possible association is indicated on the bottom part of LG3 (bright green), but it was not statistically significant since the significance threshold (6.5) was higher than in most other GWAS due to the overall very large number of SNPs in the study.

In an unpublished GWA study (Ahmadi-Afzadi, Muranty, Nybom and Durel) with lesion size data collected for 180 *P. expansum*-inoculated, mostly N. European apple cultivars, a not fully significant association with lesion decay was found on the bottom part of LG3 with the above-mentioned Axiom^®^Apple 480K array (Figure 1). A considerably larger sample size is generally needed for the detection of significant SNP-based associations in unrelated material [85]. Whether this putative QTL is close to the QTL for resistance against *P. expansum* in *M. sieversii* as described by Norelli et al. [59] has not been established yet. Nevertheless, the implications are highly interesting, since co-location of these two QTLs would indicate that there is considerable variation for *P. expansum* resistance not just in the wild species of *M. sieversii*, but also in *M.* × *domestica*.

Nowadays, genes can also be located using the increasingly affordable methods for whole-genome re-sequencing. Together with bulked segregant analysis, this type of data was used to locate genes for resistance against Glomerella leaf spot caused by *C. fructicola* [35,36]. Genome-wide comparison of SNP profiles between the resistant and the susceptible bulks, constructed from F1 individuals derived from a cross between ‘Golden Delicious’ and ‘Fuji’, enabled the fine-mapping of an *Rgls* locus on LG15. Whether markers for resistance against Glomerella leaf spot will prove to be useful for predicting resistance against storage rot on apple fruits caused by the same species remains to be seen. Previous studies have shown that at least some isolates of *C. fructicola* show clear organ specialization, with differences in their impact on the enzymatic oxidant defence system of the host [37].

## 5. Impact of Fruit Ripening Period, Fruit Texture and Chemical Contents

In this review, ‘ripening period’ refers to the date at which a particular cultivar is ready for (commercial) harvest. When many cultivars are investigated in the same study, ripening period is usually quantified as the number of days between harvest of the earliest-ripening cultivar and harvest of the cultivar in question. Comparative scoring data for cultivars grown at different sites can be obtained by defining ripening period relative to well-known major cultivars like ‘Gala’ or ‘Golden Delicious’ grown at all sites, and a correction for site and year if needed [84]. ‘Fruit maturity’ instead refers to the stage (e.g., unripe, medium ripe, overripe) that a particular fruit may have reached [86,87]. Early-ripening cultivars (summer apples) generally have high climacteric respiration and ethylene production rates and mature quickly, whereas late-ripening cultivars (autumn apples) have lower respiration and ethylene production rates and mature more slowly [4].

Determination of fruit ‘texture’ is complicated since many different variables are involved, with consumer-perceived chewiness, crunchiness, juiciness, crispness and firmness being especially important. Firmness is the most commonly investigated parameter, since it can be assessed with a simple handheld penetrometer. An automated texture analyser, measuring several texture parameters simultaneously, has been used in some recent studies. When analysing 86 apple cultivars with this device, a set of mechanically based variables showed high correspondence to ‘firmness’ while an acoustics-based set corresponded to ‘crispness’ as perceived by human senses [88]. Firmness is often measured both at harvest and after storage, since it decreases during the harvesting period, usually with a major reduction in conjunction with, or more commonly, just after the climacteric rise in IEC [4]. Commercial fruit, especially if destined for long-term storage, is usually harvested when it is in a kind of steady state, i.e., before the rise in IEC. In addition to the initial firmness, amount of firmness retained after cold storage is crucial for the choice of apple cultivars in modern orchards. Loss in firmness (difference in firmness between measurements) is termed fruit softening and is sometimes divided by number of weeks in storage to yield ‘softening rate’, which enables comparisons to be made among cultivars stored for different periods of time [78]. Large-scale screenings of Swedish and Norwegian cultivar collections have revealed positive associations between ripening period with firmness at harvest (early-ripening cultivars are less firm at harvest compared to late-ripening cultivars) and negative associations between ripening time and softening rate (early-ripening cultivars lose firmness faster than late-ripening cultivars) [15,78]. By contrast, no correlation was found between commercial harvest date (i.e., ripening period) and fruit firmness when 23 Belgian cultivars were compared [8].

Amounts of various chemical compounds, both in the flesh and in the peel, are to a large extent affected by environmental factors like soil, pruning, fertilizer, irrigation and weather conditions [4,89]. To the extent that chemical contents also differ genetically among cultivars, genotypes with high contents of beneficial compounds could become very valuable in breeding for resistance. Quantification of chemical contents in a set of different apple genotypes is however quite complicated, and evaluations must be made at comparable stages of fruit maturation. Sugars (often estimated as soluble solids content, SSC) increase considerably in the fruit flesh due to starch hydrolysis during the ripening process, while acids (often estimated as titratable acidity, TA) decrease through respiratory metabolism [4]. The ripening behaviour of the studied cultivars may also interfere; fruit of early-ripening cultivars have been noted for relatively low levels of ascorbate at harvest and a more rapid reduction during the following 10-day period compared to fruit of late-ripening cultivars [8]. In addition, chemical contents in the fruit commonly change as a response to infection; different species of fungi acidify or alkalify their environment to facilitate infection and necrotrophic growth in the fruit [14]. Sugars, i.e., mainly sucrose in apples, have an important role in respiration and energy consumption which increases after infection. For the defence mechanisms, various phenolic substances may, however, be the most important.

## 6. Possible Associations between Fruit Ripening and Resistance

Wound inoculations were carried out in a set of 92 Swedish-grown apple cultivars in 2010 and in a set of 45 cultivars in 2012, with 45 fruits inoculated for each cultivar [5]. The fruits were all picked at a maturity stage corresponding to commercial harvest date, and had been stored for 6 (early-ripening cultivars) or 12 weeks (late-ripening). A significant negative correlation was found between lesion diameter and harvest date (i.e., ripening period) for both early-ripening and late-ripening cultivars in 2010, but only for late-ripening cultivars in 2012. In a similar study of 45 cultivars grown in Sweden and 45 cultivars grown in Norway, investigated in 2012 and 2013, lesion diameter was again negatively correlated with ripening period [15]. As expected, lesion diameter was negatively correlated with firmness at harvest, and positively correlated with fruit softening in both of these studies [5,15]. A negative association was similarly found between fruit firmness and lesion diameter in a study of Iranian cultivars [58]. The relationship between firmness and lesion decay reported here is probably associated with the ability of cell walls to withstand attacks from pectolytic enzymes of *P. expansum*. Susceptibility to another wound-infecting species, *B. cinerea,* similarly decreased with later commercial harvest period (i.e., high values for ripening period) in a set of 23 Belgian-grown cultivars [8]. The basis of this relationship is not known, but late-ripening cultivars tend to have firmer fruit and higher levels of antioxidants and ascorbate, among other compounds, both of which may improve resistance against fungi.

Wound inoculations carried out with *C. gloeosporioides* in a set of 70 Swedish-grown cultivars demonstrated a negative association between lesion diameter and ripening period in early-ripening cultivars but not in late-ripening [5], and also not for *C. acutatum* [27] (fruits of 18 cultivars inoculated by attaching a tube with inoculum) or *C. gloeosporioides* [8] (Gloeosporium rot; fruits of 23 cultivars dipped in a spore solution). Presently, it is not possible to determine whether the inconsistency between studies is caused by differences in inoculum or inoculation method, for example, or the low number of sampled cultivars in some of the studies. Cultivars assessed from natural infection to be resistant to the lenticel-infecting *Pezicula alba* (*Neofabraea alba)* had, on average, firmer flesh than susceptible cultivars, but considerable overlap was noted [40]. A corresponding relationship was found also when wound-inoculating 9 apple cultivars with *C. fioriniae* [30], but not when non-wound-inoculating 18 cultivars with *C. acutatum* [27]. Possibly, the ability to withstand pectolytic enzymes is overshadowed by parameters involved in fungal entry for non-wound-inoculated fruit.

Data have been collected for both chemical contents and lesion diameter after wound inoculation with *P. expansum* in a number of different apple cultivars, and/or germplasm of the wild species *M. sieversii*. In some of these studies, chemical analyses were executed only on healthy fruit, while both healthy and infected fruit were analysed in other studies. Associations between chemical compounds and other storage rots like *B. cinerea* and *Colletotrichum* spp. have also been investigated [8,30].

Content of TA (or malic acid, which constitutes the major portion of acids in apple) appears to be lower in *P. expansum*-inoculated fruits compared to control fruit [6,63,90]. The reason for this has not been determined, but fungal acidification seems to be mediated mainly by gluconic acid (12, 32, 55]. To what extent—if any—variation in TA of healthy fruit affects susceptibility is not clear. Weak, but still significant, negative correlations between TA in the fruit flesh at harvest and susceptibility to *P. expansum* were reported in 83 *M. sieversii* accessions [56], and in 43 Iranian-grown cultivars [58], whereas no correlations were found in analyses of the peel and flesh of 24 Swedish-grown apple cultivars [6], or in another set with 10 Swedish-grown cultivars [60]. There have been only a few corresponding studies on other fungi, but Davey et al. [8] reported a negative correlation between TA and lesions caused by inoculation with *B. cinerea*, and Blažek et al. [40] found a similarly negative association between TA in apple juice samples and lesions caused by storage rots in general. Although these negative correlations could be an indicator of resistance, they may also stem from a somewhat uneven level of maturity in the sampled genotypes; less mature and therefore comparatively sour apples are usually more resistant to storage rots.

The relationship between SSC in healthy fruit and lesion diameter after inoculation with *P. expansum* is also unclear. Naeem-Abadi et al. [58] reported of a weak negative correlation between lesion diameter and SSC, while Tahir et al. [15] did not find any correlation in two sets of 45 cultivars each (grown in Norway and Sweden, respectively). Janisiewicz et al. [56] instead reported of a weak positive correlation, and concluded that a high sugar content might contribute to the susceptibility of fruit to decay. SSC content in juice of 10 apple cultivars was also positively correlated with lesion diameter [60]. Similarly, LEGR (lesion expansion growth rate) from wound-inoculation of nine apple cultivars with *C. fioriniae* was positively associated with SSC [30]. However, the association may be affected by biased sampling, since a somewhat over-mature fruit tends to be sweeter and also more susceptible to storage rots.

An important role of polyphenols in the defence against *P. expansum* was deduced from a study showing that three relatively resistant *M. sieversii* accessions had higher concentrations of procyanidins, dihydrochalcone, flavonols and hydroxycinnamic acids compared to ‘Golden Delicious’ and a susceptible accession of *M. sieversii* [61]. In a larger study, contents of total phenols (TPH) and of 10 individual phenolic compounds were quantified in peel and flesh fractions of both control and *P. expansum*-inoculated fruits of 24 apple cultivars grown in Sweden [6]. Contents in the peel were 4–32 times higher (depending on cultivar) compared to the flesh, and were usually lower in inoculated fruits compared to healthy fruits. Correlation analysis revealed a significant association between lesion diameter in inoculated fruits and TPH in the peel, as well as various flavonols (quercetin compounds) and procyanidin B2. Analyses carried out on healthy control fruits of the same cultivars yielded fewer but still significant associations with the flavonol content, suggesting that pre-formed polyphenolic compounds may inhibit or decrease the development of disease in fruits upon fungal attack. By contrast, no associations were found involving the dihydrochalcone phloridzin, and only one significant association involving hydroxycinnamic acids, namely in the flesh of control fruits. In another study with 10 Swedish-grown cultivars, TPH as well as seven individual polyphenolic compounds were, as expected, overall much higher in the fruit of five cider apple cultivars originating from a recent breeding program at Long Ashton, UK, compared to fruit of five table apple cultivars. However, contrary to expectations, inoculation with *P. expansum* produced larger lesions in all of the cider cultivars compared to the table cultivars. In addition, significant positive correlations were found between lesion diameter and content of procyanidin B2, epicatechin and trimer aglycone. Since all five cider apple cultivars were offspring of the relatively susceptible table apple ‘James Grieve’, they may perhaps have inherited a genetically determined susceptibility unrelated to chemical contents.

Apple cultivars with comparatively high contents of anthocyanin and ascorbic acid showed less damage caused by *Neofabraea* spp. compared to cultivars with lower contents [47]. Similarly, higher contents of phenolic compounds were noted in both skin and flesh of comparatively resistant cultivars grown in the Czech Republic, while lower contents were found in cultivars susceptible to various storage rots [40]. The more resistant cultivars also showed overall higher contents of epicatechin, catechin and chlorogenic acid in the skin.

Chromosomes 3, 10 and 16 have shown associations with ripening period in progeny-based linkage-mapping studies [91,92,93,94,95]. In addition, a QTL on LG9 controls the tight linkage between early ripening and red flesh/red leaves [96]. By contrast, Migicovsky et al. [97] did not find any associations with harvest period on LG10 and LG16 in a GWAS based on single-locus tests, but identified associations with two SNPs on LG3. In the largest study so far, almost 1200 apple cultivars from six European germplasm collections were screened with the Axiom^®^Apple 480K array [32]. This work identified in six SNPs with strong associations to ripening period: four on the bottom part of LG3, one on the bottom part of LG10 and one on the top of LG16. A number of candidate genes were located within the confidence intervals of these genomic regions, suggesting that transcription factors such as MADS- and NAC-containing genes have a major role. Whether the QTL on LG3 found by Norelli et al. [59] for resistance in *M. sieversii* against *P. expansum*, and the possible QTL for resistance among *M.* × *domestica* cultivars (Ahmadi-Afzadi, Muranty, Nybom and Durel, unpublished) have any connections with the QTL for ripening time found on LG3 [84,97] is not yet known.

Variation in fruit-texture parameters is apparently also under the control of multiple genes, affecting fruits at harvest and/or after storage, including *MdACS1* and *MdACO1,* which have been mapped to LG15 and LG10, respectively [77,98]. A direct effect of ethylene on fruit texture is the regulation of enzymatic breakdown of cell walls and middle lamellae in the fruit. The biallelic endopolygalacturonase gene *MdPG1,* which affects fruit softening during ripening and cold storage, has also been mapped to LG10, at a distance of 37 cm from *MdACO1* [98,99,100]. As far as we know, there have been no reports to date of significant associations between the allelic composition in any of these fruit-texture genes and susceptibility to the different storage-rot diseases.

A number of loci that affect chemical contents have been located on the apple genome, including *Ma1* on LG16 for acidity [101]. One important QTL controlling phenolic compounds is co-located with *MdLAR1* (leucoanthocyanidin reductase 1) on LG16, which affects acidity, bitter pit and fruit cracking [7,22]. In a recent study, 82 Swedish-grown apple cultivars previously phenotyped for fruit firmness, fruit softening rate and lesion diameter after wound inoculation with *P. expansum*, were subsequently screened by Taqman^®^ and high-resolution melting (HRM) assays for 15 qPCR-based molecular markers [102]. These markers target loci linked to fruit texture and chemical composition on LG10 and LG16, and derive from the IRSCOA v1.0 developed by the RosBREED SNP Consortium [98]. The results revealed significant phenotype–genotype associations between two SNPs in the *MdLAR1* locus (ss475881696 and ss475882555) and lesion diameter. Interestingly, Chagné et al. [98] reported these two SNP markers to be associated with both fruit acidity and fruit firmness and crispness. In addition, a non-significant relationship (*p* = 0.08) was indicated between fruit softening rate in the 82 Swedish cultivars and two SNPs in the *Ma1* locus (ss475876558 and Ma1-SNP1455).

In parallel with the above-mentioned HRM analysis with 15 qPCR-based markers [102], association analyses were also conducted for chemical data previously collected on 20 apple cultivars (Ahmadi-Afzadi, Nybom, Kirk, and Chagné, unpublished data). Content of procyanidins in the fruit flesh was significantly associated with two loci (ss475883942 and ss475881704) on LG16, as was content of chlorogenic acid with another two loci (*Ma1-SNP1455* and ss475883359) on LG16. Contents of several flavonols (quercetins) were also associated with the locus FEM-cg-19 on LG10. However, further studies with larger sample sizes are needed.

## 7. Conclusions and Perspectives

For necrotrophic storage rots like blue mould caused by *P. expansum*, animal- or man-made wounds in the fruit are major entry points. Wound inoculation with fungal spores can therefore produce relevant assessments of cultivar resistance. *Penicillium expansum* has been hypothesized to counteract the defence mechanisms of the fruit by manipulating its endogenous ethylene biosynthesis. This fungus also lowers the pH of the host tissue and secretes lytic enzymes that promote the degradation of cell walls. Consequently, late-ripening apple cultivars, which are often characterized by a lower ethylene climacteric burst and higher fruit firmness, seem to fare better than early-ripening cultivars when attacked by blue mould. Associations between lesion decay and chemical contents of the fruit are, overall, less clear-cut, but indicate that baseline contents of especially phenolic substances, as well as changes in contents caused by infection, have an effect on level of resistance. Much less information is available for other necrotrophs, like *Botrytis* and *Monilinia*, but resistance against these fungi is most likely similarly related to fruit-ripening behaviour.

Biotrophic storage rots like *Colletotrichum* and *Neofabraea* enter mainly through open flower calyces and/or through lenticels and micro-cracks in the developing fruit. Wound inoculations therefore measure only one part of the heritable resistance in these fungi. Unfortunately, non-wound-inoculation methods are more time-consuming to apply, and the resulting damage is more difficult to quantify. Due to the resulting low number of in-depth studies of these fungi, the impact of ethylene-related processes on resistance against biotrophic storage rots cannot yet be ascertained. So far, however, associations with ripening period and fruit firmness appear to be less clear-cut for *Colletotrichum* and *Neofabraea* compared to the better-studied *P. expansum*.

For plant breeders, superior germplasm with well-characterized major-impact genes, preferably provided with easy-to-use DNA markers, is number one on the wish-list. Optimal development of storage-rot-resistant apple cultivars would require a geographically very diverse germplasm in order to cover the large variation in ripening-period-related traits. Superior inoculation methods must therefore be developed in order to produce large sets of phenotypic data to act as a basis for genetic analyses.

## Figures and Tables

**Table 1:** Fungi commonly reported to cause storage rots in apple fruits.

Taxon	Synonyms	Popular Name	References
*Alternaria* spp., e.g., *A. alternata* (Fr.) Keissl. and *A. tenuissisima* (Kunze) Wiltshire		Alternaria rot, calyx-end rot	[3,16,20,21]
*Botryosphaeria* spp., e.g., *B. dothidea* (Moug. ex Fr.) Ces. & De Not. and *B. obtusa* (Schwein.) Shoemaker	*Diplodia seriata* De Not., *Sphaeropsis malorum*	Black rot (*B. obtusa*), white rot (*B. dothidea*)	[20,21,22]
*Botrytis cinerea* Pers.		Grey mould, dry-eye rot	[8,16,21,23,24,25,26]
*Cadophora* spp., e.g., *C. luteo-olivacea* (J.F.H. Beyma) T.C. Harr. & McNew and *C. malorum* (Kidd & Beaumont) W. Gams	*Phialophora luteo-olivacea* J.F.H. Beyma and *P. malorum* (Kidd & Beaumont) McColloch, respectively	Side rot	[16,20]
*Colletotrichum acutatum* J.H. Simmonds incl. e.g., *C. fioriniae* Marcelino & Gouli ex R.G. Shivas & Y.P. Tan	*Glomerella acutata* Guerber & J.C. Correll	Bitter rot (Glomerella leaf spot on leaves)	[9,27,28,29,30,31]
*Colletotrichum gloeosporioides* (Penz) Penz. & Sack. incl. e.g., *C. fructicola* Prihastuti, L. Cai & K.D. Hyde	*Glomerella cingulata* (Stoneman) Spauld. & H. Schrenk, *Gloeosporium fructigenum* Berk., *Colletotrichum fructigenum* (Berk.) Vassiljevsky	Bitter rot (Glomerella leaf spot on leaves)	[5,8,9,14,16,29,32,33,34,35,36,37]
*Fusarium* spp., e.g., *F. avenaceum* (Fr.) Sac., *F. lateritium* Nees ex. Fr. and *F. proliferatum*		Fusarium rot, wet core rot	[3,16,20,21]
*Monilinia fructigena* (Aderh. & Ruehl.) Honey ex Whetzel	*Monilia fructigena* (Pers.) Pers.	Brown rot	[16,19,21,38]
*Monilinia laxa* (Aderh. & Ruhland) Honey	*Monilia cinerea* Bonord.	Brown rot	[21]
*Mucor piriformis* A. Fisch.		Mucor rot	[20,21,25]
*Neofabraea actinidiae* (P.R. Johnst., M.A. Manning & X. Meier) P.R. Johnst.	*Cryptosporiopsis actinidiae* P.R. Johnst., M.A. Manning & X. Meier	Bull’s eye rot, lenticel rot	[39]
*Neofabraea alba* (E.J. Guthrie) Verkley	*Gloeosporium album* Osterw., *Pezicula alba* E.J. Guthrie, *Phlyctema vagabunda* Desm., *Neofabraea vagabunda* Desm. (P.R. Johnst.)	Bull’s eye rot, lenticel rot	[3,11,16,28,40,41,42,43,44,45]
*Neofabraea brasiliensis* Sanhueza & Bogo		Bull’s eye rot, lenticel rot	[39]
*Neofabraea kienholzii* (Seifert, Spotts & Lévesque) Spotts, Lévesque & Seifert	*Cryptosporiopsis kienholzii* Seifert, Spotts & Lévesque	Bull’s eye rot, lenticel rot	[17,46]
*Neofabraea malicorticis* (Cordley) H.S. Jacks.	*Gloeosporium malicorticis* Cordley, *Pezicula malicorticis* (Cordley) Nannf., *Cryptosporiopsis malicorticis* (Cordley) Nannf.	Bull’s eye rot, lenticel rot	[16,25,43,44,45]
*Neofabraea perennans* Kienholz	*Gloeosporium perennans* Zeller & Childs, *Pezicula perennans* (Kienholz) Dugan, R.G. Roberts & G.G. Grove	Bull’s eye rot, lenticel rot	[44,45,46,47,48,49]
*Neonectria ditissima* (Tul. & C. Tul.) Samuels & Rossman	*Nectria ditissima* Tul. & C. Tul., *Nectria galligena* Bres.	Nectria rot (apple canker or European canker on trees)	[20,50]
*Penicillium expansum* Link		Blue mould	[3,5,6,12,15,21,25,31,32,33,38,51,52,53,54,55,56,57,58,59,60,61,62,63,64,65,66]
*Phacidiopycnis* spp., e.g., *P. malorum* Potebnia and *P. washingtonensis* Xiao & J.D. Rogers	*Potebniamyces pyri* (Berk. & Broome) Dennis	Phacidiopycnis rot	[20]

**Table 2:** Infection mode and period of infection for some storage rots in apple fruits.

Taxon	Mode of Infection	Period of Infection
*Alternaria* spp.	Mainly through open calyces	Developing fruit in the orchard
*Botryosphaeria* spp.	Mainly through lenticels or micro-cracks	Developing fruit in the orchard
*Botrytis cinerea*	Mainly through wounds caused by animals or humans	Mainly during harvest and handling operations
*Cadophora* spp.	Mainly through lenticels or micro-cracks	Developing fruit in the orchard
*Colletotrichum* spp.	Mainly through lenticels or micro-cracks	Developing fruit in the orchard
*Fusarium* spp.	Mainly through wounds or open calyces	Developing fruit in the orchard
*Monilinia* spp.	Mainly through wounds caused by animals or humans	Developing fruit and during harvest and handling operations
*Mucor pyriformis*	Through micro-cracks or wounds, often at stem or calyx end of fruit	Developing fruit (last month) or during harvest and handling operations
*Neofabraea* spp.	Mainly through lenticels or micro-cracks	Developing fruit in the orchard
*Neonectria ditissima*	Mainly through wounds or at calyx end of fruit	During and just after flowering (eye rot) and developing fruit (storage rot)
*Penicillium expansum*	Mainly through wounds caused by animals or humans	Mainly during harvest and handling operations
*Phacidiopycnis* spp.	Mainly at stem end or at calyx end, sometimes wound-mediated	Developing fruit in the orchard

**Table 3:** Inoculation methods for quantification of damage caused by storage rots.

Method	Fungi	References
*On trees—spores*		
Cheesecloth strips: strips soaked in spore suspension applied to fruit in orchard 3–4 weeks prior to harvest	*Colletotrichum acutatum*	[27]
Spraying: spore suspension sprayed onto fruit in orchard; most efficient towards end of growing season	*Neofabraea* spp.	[46]
Spraying: spore suspension sprayed onto flowers and fruit on potted trees	*Neonectria ditissima*	[50]
*Detached fruit—spores*		
Agar plate: intact fruit placed on petri plate with agar and a droplet of spore suspension	*C. acutatum*	[67]
Attached tubes: spore-suspension-containing microcentrifuge tubes attached to fruit with modelling clay	*C. acutatum*	[27]
Dipping: intact fruit dipped in spore solution	*C. gloeosporioides*	[8]
Spraying: spore suspension sprayed onto intact fruit	*C. fioriniae*	[30]
	*Neofabraea alba*	[43]
Water agar: intact fruit placed on water agar with a droplet of spore suspension	*N. alba*	[28]
Wound inoculation: spore solution deposited into 1–5 holes made by a nail or a pipette tip in the fruit skin	*Penicillium expansum*	[46,56]
	*Botrytis cinerea*	[8]
	*Colletotrichum* spp.	[27,30]
	*Neofabraea* spp.	[25,49]
*Detached fruit—mycelium*		
Mycelial discs: attachment of five 7 mm large discs onto the fruit using adhesive tape	*Botryosphaeria dothidea*	[22]
Mycelial plugs: plugs inserted into fruit flesh, sometimes under the skin of the fruit	*Neofabraea* spp.	[39,40,45]
	*B. cinerea*	[26]
